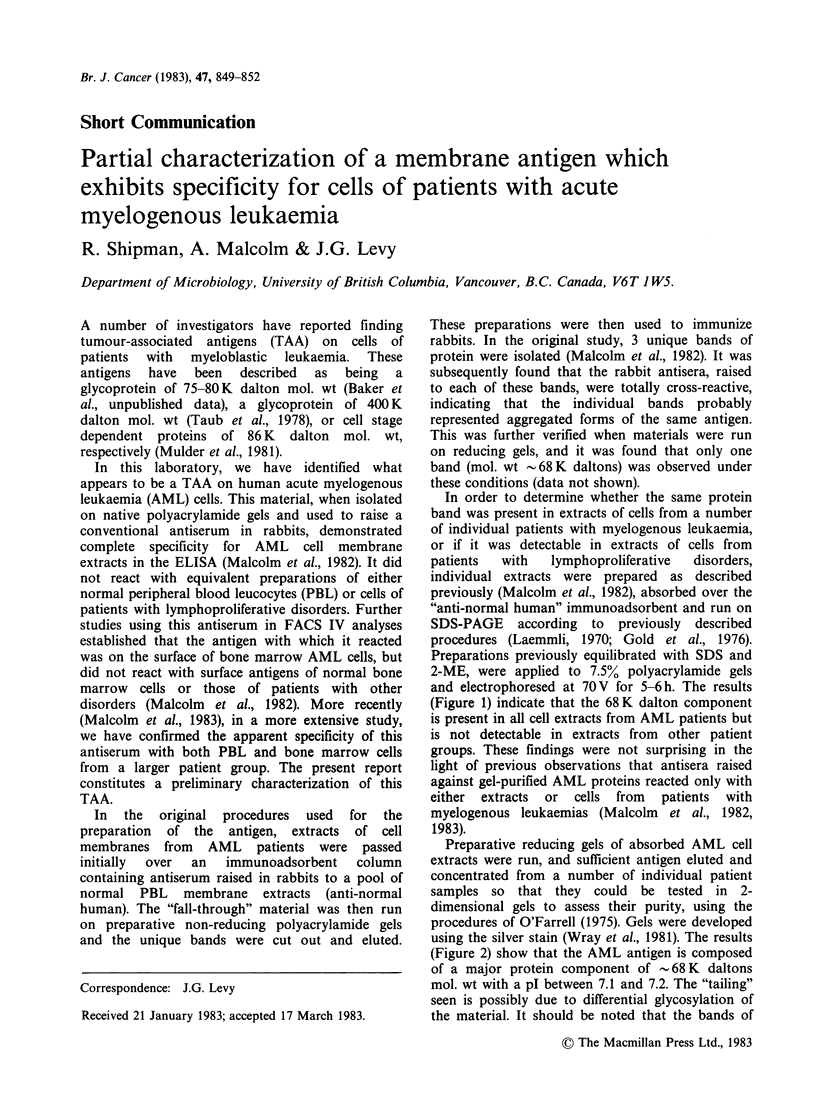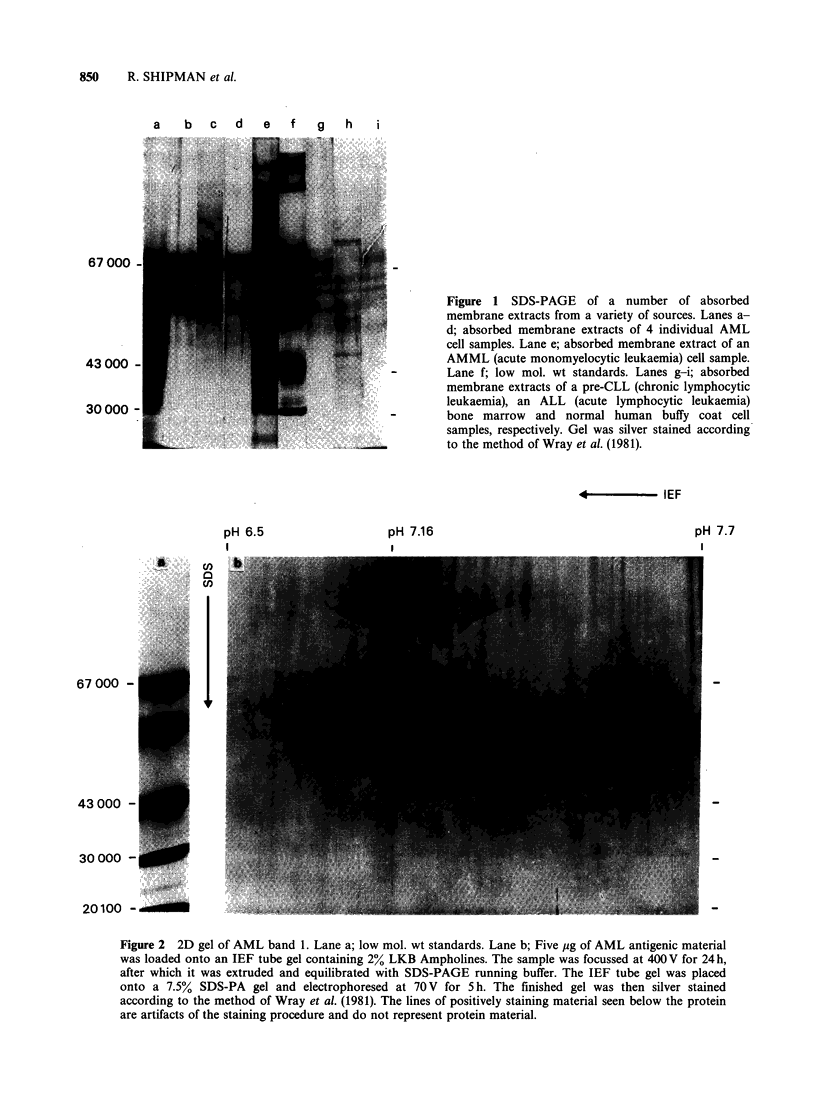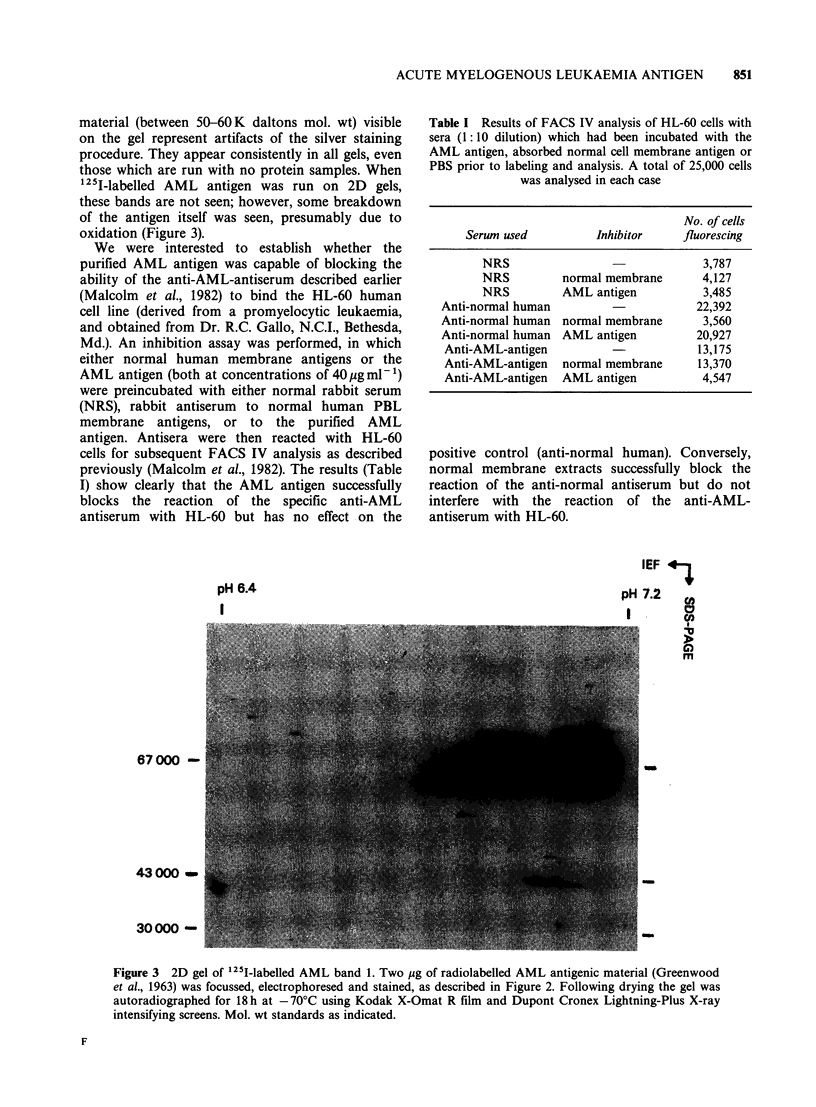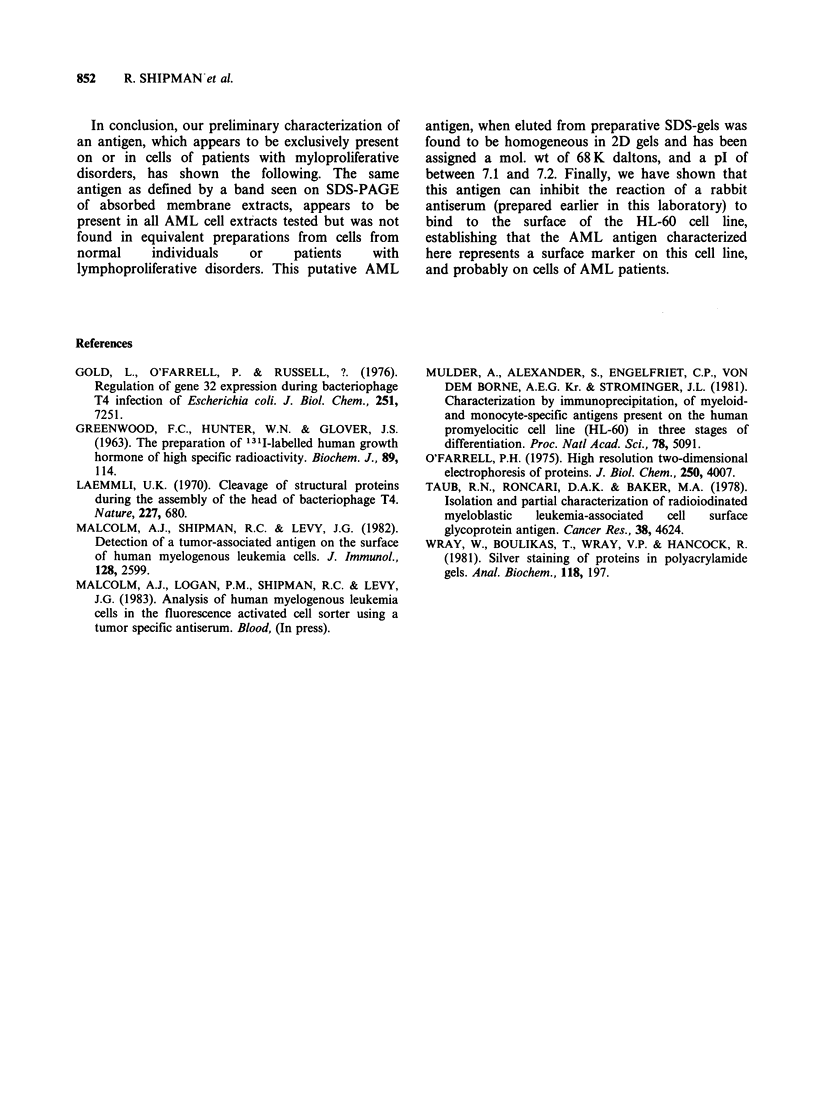# Partial characterization of a membrane antigen which exhibits specificity for cells of patients with acute myelogenous leukaemia.

**DOI:** 10.1038/bjc.1983.140

**Published:** 1983-06

**Authors:** R. Shipman, A. Malcolm, J. G. Levy

## Abstract

**Images:**


Br. J. Cancer (1983), 47, 849–852

**Short Communication**

### Partial characterization of a membrane antigen which exhibits specificity for cells of patients with acute myelogenous leukaemia

R. Shipman, A. Malcolm & J.G. Levy

*Department of Microbiology, University of British Columbia, Vancouver, B.C. Canada, V6T 1W5.*

A number of investigators have reported finding tumour-associated antigens (TAA) on cells of patients with myeloblastic leukaemia. These antigens have been described as being a glycoprotein of 75–80 K dalton mol. wt (Baker *et al.*, unpublished data), a glycoprotein of 400 K dalton mol. wt (Taub *et al.*, 1978), or cell stage dependent proteins of 86 K dalton mol. wt, respectively (Mulder *et al.*, 1981).

In this laboratory, we have identified what appears to be a TAA on human acute myelogenous leukaemia (AML) cells. This material, when isolated on native polyacrylamide gels and used to raise a conventional antiserum in rabbits, demonstrated complete specificity for AML cell membrane extracts in the ELISA (Malcolm *et al.*, 1982). It did not react with equivalent preparations of either normal peripheral blood leucocytes (PBL) or cells of patients with lymphoproliferative disorders. Further studies using this antiserum in FACS IV analyses established that the antigen with which it reacted was on the surface of bone marrow AML cells, but did not react with surface antigens of normal bone marrow cells or those of patients with other disorders (Malcolm *et al.*, 1982). More recently (Malcolm *et al.*, 1983), in a more extensive study, we have confirmed the apparent specificity of this antiserum with both PBL and bone marrow cells from a larger patient group. The present report constitutes a preliminary characterization of this TAA.

In the original procedures used for the preparation of the antigen, extracts of cell membranes from AML patients were passed initially over an immunoadsorbent column containing antiserum raised in rabbits to a pool of normal PBL membrane extracts (anti-normal human). The "fall-through" material was then run on preparative non-reducing polyacrylamide gels and the unique bands were cut out and eluted. These preparations were then used to immunize rabbits. In the original study, 3 unique bands of protein were isolated (Malcolm *et al.*, 1982). It was subsequently found that the rabbit antisera, raised to each of these bands, were totally cross-reactive, indicating that the individual bands probably represented aggregated forms of the same antigen. This was further verified when materials were run on reducing gels, and it was found that only one band (mol. wt $\sim$68 K daltons) was observed under these conditions (data not shown).

In order to determine whether the same protein band was present in extracts of cells from a number of individual patients with myelogenous leukaemia, or if it was detectable in extracts of cells from patients with lymphoproliferative disorders, individual extracts were prepared as described previously (Malcolm *et al.*, 1982), absorbed over the "anti-normal human" immunoadsorbent and run on SDS-PAGE according to previously described procedures (Laemmli, 1970; Gold *et al.*, 1976). Preparations previously equilibrated with SDS and 2-ME, were applied to 7.5% polyacrylamide gels and electrophoresed at 70 V for 5–6 h. The results (Figure 1) indicate that the 68 K dalton component is present in all cell extracts from AML patients but is not detectable in extracts from other patient groups. These findings were not surprising in the light of previous observations that antisera raised against gel-purified AML proteins reacted only with either extracts or cells from patients with myelogenous leukaemias (Malcolm *et al.*, 1982, 1983).

Preparative reducing gels of absorbed AML cell extracts were run, and sufficient antigen eluted and concentrated from a number of individual patient samples so that they could be tested in 2-dimensional gels to assess their purity, using the procedures of O'Farrell (1975). Gels were developed using the silver stain (Wray *et al.*, 1981). The results (Figure 2) show that the AML antigen is composed of a major protein component of $\sim$68 K daltons mol. wt with a pI between 7.1 and 7.2. The "tailing" seen is possibly due to differential glycosylation of the material. It should be noted that the bands of

Correspondence: J.G. Levy

Received 21 January 1983; accepted 17 March 1983.

© The Macmillan Press Ltd., 1983

**Figure 1** SDS-PAGE of a number of absorbed membrane extracts from a variety of sources. Lanes a–d; absorbed membrane extracts of 4 individual AML cell samples. Lane e; absorbed membrane extract of an AMML (acute monomyelocytic leukaemia) cell sample. Lane f; low mol. wt standards. Lanes g–i; absorbed membrane extracts of a pre-CLL (chronic lymphocytic leukaemia), an ALL (acute lymphocytic leukaemia) bone marrow and normal human buffy coat cell samples, respectively. Gel was silver stained according to the method of Wray et al. (1981).

**Figure 2** 2D gel of AML band 1. Lane a; low mol. wt standards. Lane b; Five $\mu$g of AML antigenic material was loaded onto an IEF tube gel containing 2% LKB Ampholines. The sample was focussed at 400 V for 24 h, after which it was extruded and equilibrated with SDS-PAGE running buffer. The IEF tube gel was placed onto a 7.5% SDS-PA gel and electrophoresed at 70 V for 5 h. The finished gel was then silver stained according to the method of Wray et al. (1981). The lines of positively staining material seen below the protein are artifacts of the staining procedure and do not represent protein material.

material (between 50–60 K daltons mol. wt) visible on the gel represent artifacts of the silver staining procedure. They appear consistently in all gels, even those which are run with no protein samples. When $^{125}$I-labelled AML antigen was run on 2D gels, these bands are not seen; however, some breakdown of the antigen itself was seen, presumably due to oxidation (Figure 3).

We were interested to establish whether the purified AML antigen was capable of blocking the ability of the anti-AML-antiserum described earlier (Malcolm *et al.*, 1982) to bind the HL-60 human cell line (derived from a promyelocytic leukaemia, and obtained from Dr. R.C. Gallo, N.C.I., Bethesda, Md.). An inhibition assay was performed, in which either normal human membrane antigens or the AML antigen (both at concentrations of 40 $\mu$g ml$^{-1}$) were preincubated with either normal rabbit serum (NRS), rabbit antiserum to normal human PBL membrane antigens, or to the purified AML antigen. Antisera were then reacted with HL-60 cells for subsequent FACS IV analysis as described previously (Malcolm *et al.*, 1982). The results (Table I) show clearly that the AML antigen successfully blocks the reaction of the specific anti-AML antiserum with HL-60 but has no effect on the positive control (anti-normal human). Conversely, normal membrane extracts successfully block the reaction of the anti-normal antiserum but do not interfere with the reaction of the anti-AML-antiserum with HL-60.

**Table I** Results of FACS IV analysis of HL-60 cells with sera (1 : 10 dilution) which had been incubated with the AML antigen, absorbed normal cell membrane antigen or PBS prior to labeling and analysis. A total of 25,000 cells was analysed in each case

| *Serum used* | *Inhibitor* | *No. of cells fluorescing* |
|---|---|---|
| NRS | — | 3,787 |
| NRS | normal membrane | 4,127 |
| NRS | AML antigen | 3,485 |
| Anti-normal human | — | 22,392 |
| Anti-normal human | normal membrane | 3,560 |
| Anti-normal human | AML antigen | 20,927 |
| Anti-AML-antigen | — | 13,175 |
| Anti-AML-antigen | normal membrane | 13,370 |
| Anti-AML-antigen | AML antigen | 4,547 |

**Figure 3** 2D gel of $^{125}$I-labelled AML band 1. Two $\mu$g of radiolabelled AML antigenic material (Greenwood *et al.*, 1963) was focussed, electrophoresed and stained, as described in Figure 2. Following drying the gel was autoradiographed for 18 h at −70°C using Kodak X-Omat R film and Dupont Cronex Lightning-Plus X-ray intensifying screens. Mol. wt standards as indicated.

In conclusion, our preliminary characterization of an antigen, which appears to be exclusively present on or in cells of patients with myloproliferative disorders, has shown the following. The same antigen as defined by a band seen on SDS-PAGE of absorbed membrane extracts, appears to be present in all AML cell extracts tested but was not found in equivalent preparations from cells from normal individuals or patients with lymphoproliferative disorders. This putative AML antigen, when eluted from preparative SDS-gels was found to be homogeneous in 2D gels and has been assigned a mol. wt of 68 K daltons, and a pI of between 7.1 and 7.2. Finally, we have shown that this antigen can inhibit the reaction of a rabbit antiserum (prepared earlier in this laboratory) to bind to the surface of the HL-60 cell line, establishing that the AML antigen characterized here represents a surface marker on this cell line, and probably on cells of AML patients.

### References

GOLD, L., O'FARRELL, P. & RUSSELL, ?. (1976). Regulation of gene 32 expression during bacteriophage T4 infection of *Escherichia coli*. *J. Biol. Chem.*, **251**, 7251.

GREENWOOD, F.C., HUNTER, W.N. & GLOVER, J.S. (1963). The preparation of $^{131}$I-labelled human growth hormone of high specific radioactivity. *Biochem. J.*, **89**, 114.

LAEMMLI, U.K. (1970). Cleavage of structural proteins during the assembly of the head of bacteriophage T4. *Nature*, **227**, 680.

MALCOLM, A.J., SHIPMAN, R.C. & LEVY, J.G. (1982). Detection of a tumor-associated antigen on the surface of human myelogenous leukemia cells. *J. Immunol.*, **128**, 2599.

MALCOLM, A.J., LOGAN, P.M., SHIPMAN, R.C. & LEVY, J.G. (1983). Analysis of human myelogenous leukemia cells in the fluorescence activated cell sorter using a tumor specific antiserum. *Blood*, (In press).

MULDER, A., ALEXANDER, S., ENGELFRIET, C.P., VON DEM BORNE, A.E.G. Kr. & STROMINGER, J.L. (1981). Characterization by immunoprecipitation, of myeloid- and monocyte-specific antigens present on the human promyelocitic cell line (HL-60) in three stages of differentiation. *Proc. Natl Acad. Sci.*, **78**, 5091.

O'FARRELL, P.H. (1975). High resolution two-dimensional electrophoresis of proteins. *J. Biol. Chem.*, **250**, 4007.

TAUB, R.N., RONCARI, D.A.K. & BAKER, M.A. (1978). Isolation and partial characterization of radioiodinated myeloblastic leukemia-associated cell surface glycoprotein antigen. *Cancer Res.*, **38**, 4624.

WRAY, W., BOULIKAS, T., WRAY, V.P. & HANCOCK, R. (1981). Silver staining of proteins in polyacrylamide gels. *Anal. Biochem.*, **118**, 197.